# Synthesis and Application of New Amphiphilic Asphaltene Ionic Liquid Polymers to Demulsify Arabic Heavy Petroleum Crude Oil Emulsions

**DOI:** 10.3390/polym12061273

**Published:** 2020-06-02

**Authors:** Ali I. Ismail, Ayman M. Atta, Mohamed El-Newehy, Mohamed E. El-Hefnawy

**Affiliations:** 1 Department of Chemistry, Rabigh College of Arts and Sciences, King Abdulaziz University, Jeddah 21589, Saudi Arabia; malhefnawy@kau.edu.sa; 2 Department of Chemistry, College of Science, King Saud University, Riyadh 11451, Saudi Arabia; melnewehy@ksu.edu.sa; 3 Department of Chemistry, Faculty of Science, Tanta University, Tanta 31527, Egypt

**Keywords:** asphaltenes, poly (ionic liquids), petroleum emulsion, demulsifiers, quaternary pyridinium cations

## Abstract

Asphaltenes are heavy petroleum crude oil components which limit the production of petroleum crude oil due to their aggregation and their stabilization for all petroleum crude oil water emulsions. The present study aimed to modify the chemical structures of isolated asphaltenes by converting them into amphiphilic polymers containing ionic liquid moieties (PILs) to demulsify the emulsion and replace the asphaltene layers surrounding the oil or water droplets in petroleum crude oil emulsions. The literature survey indicated that no modification occurred to produce the PILs from the asphaltenes. In this respect, the asphaltenes were modified via oxidation of the lower aliphatic chain through carboxylation followed by conversion to asphaltene acid chloride that reacted with ethoxylated N-alkyl pyridinium derivatives. Moreover, the carboxylation of asphaltenes was carried out through the Diels–Alder reaction with maleic anhydride that was linked with ethoxylated N-alkyl pyridinium derivatives to produce amphiphilic asphaltene PILs. The produced PILs from asphaltenes acid chloride and maleic anhydride were designated as AIL and AIL-2. The chemical structure and thermal stability of the polymeric asphaltene ionic liquids were evaluated. The modified structure of asphaltenes AIL and AIL-2 exhibited different thermal characteristics involving glass transition temperatures (T_g_) at −68 °C and −45 °C, respectively. The new asphaltenes ionic liquids were adsorbed at the asphaltenes surfaces to demulsify the heavy petroleum crude emulsions. The demulsification data indicated that the mixing of AIL and AIL-2 100 at different ratios with ethoxylated N-alkyl pyridinium were demulsified with 100% of the water from different compositions of O:W emulsions 50:50, 90:10, and 10:90. The demulsification times for the 50:50, 90:10, and 10:90 O:W emulsions were 120, 120, and 60 min, respectively. The interaction of the PILs with asphaltene and mechanism of demulsification was also investigated.

## 1. Introduction

Asphaltenes are the heaviest fraction of unconventional petroleum components, showing a series of one single or many crosslinked polycyclic aromatic and aliphatic groups as building blocks embedded with a trace amount of metal (V, Ni, Fe and Cu) [1]. They are contaminated as high molecular weight constituents (over a wide range from less than 10^3^ to 10^9^ g. mol^−1^) in many crude petroleum residua and bitumen (natural asphalts) [1]. The higher content of asphaltenes produces viscous heavy petroleum crude oils. It is now widely accepted that the polycondensed aromatics are linked with short alkyl groups and polar heteroatoms based on S, N, and O in functional groups such as ketone, thiophenes, pyridines, and porphyrins [1,2]. Asphaltenes have been considered as amphiphilic components with the presence of hydrophobic interactions of aliphatic chains or hydrophilic interactions of polycondensed aromatics and heteroatoms through acid-base interactions (electron donor-acceptor and hydrogen bonding). This allows the interaction with other hydrophilic and hydrophobic crude oil components or oilfield chemicals, forming stable films at oil–water or air–water interfaces [3,4,5]. The amphiphilic characteristics of asphaltenes are responsible for undesirable problems such as the formation of petroleum crude oil water emulsions that increased the crude oil viscosity owing to the formation of rigid interfacial films among water and hydrophilic moieties of asphaltenes and other crude oil fractions [6,7,8,9]. The desirable interactions of asphaltenes with the petroleum crude oil waxes or oilfield polymeric additives without aggregation or associations facilitate the followability of the petroleum crude oils in the production, transportation, and refining industries [10,11,12]. The polymer chemical structures that are used as oilfield chemicals and interacted with asphaltenes consist of two main parts: (i) organic backbones linked with polar functional groups, and (ii) hydrophobic aliphatic side chains that provide sufficient dispersion and solubility into crude oils having different carbon numbers [13]. The new technique proposed in the present work is based on the modification of asphaltene chemical structures for application as polymeric additives, increasing asphaltenes dispersion in the crude oil, and solving the asphaltene deposition and petroleum crude oil emulsion problems instead of using organic or organometallic polymers. The literature shows that there are some oxidizing agents such as permanganate, cerium, chromate, dichromate, peroxide, ozone, tetroxide, peroxy acids, halogen-containing (e.g., hypochlorite, chlorite, chlorate, perchlorate, and analogous halogen-containing compounds) and derivatives that can be used to oxidize asphaltene to produce new compatible oilfield chemicals [14,15]. Moreover, asphaltenes were reacted with phosphorous trichloride to produce a phosphochlorinated-asphaltene, as well as being modified with equimolar amounts of aliphatic or aromatic amines and polyamines for application as oilfield chemicals [16]. Moreover, the phosphochlorinated-asphaltene reacted with polypropylene oxide to produce amphiphilic surfactants that act as asphaltenes dispersants for heavy crude oil [16]. The asphaltene was also converted to anionic amphiphiles by sulfonation and used as capping for magnetite to use as oil spill collectors for heavy crude oil [17,18]. However, the preparations of amphiphilic asphaltenes ionic liquids (ILs) and polymeric surfactants were not reported elsewhere in the literature. 

Recently, ILs and poly (ionic liquids), PILs, that contain imidazolium, pyridinium, and quaternary ammonium cations having a low melting point, non-flammability, and higher thermal stability were used as green oilfield chemicals in the petroleum industry [19,20,21,22,23,24]. ILs formulations are based on amines, block copolymers, and hydrophobic ILs (1,5-dicarboxy-pentane-2-ammonium, pyridinium, isoquinolinium, imidazolium, ammonium and ammonium carboxymethane, and an anion selected from the group consisting of R_5_COO^−^, Cl^−^, Br^−^, [BF_4_]^−^, [PF_6_]^−^, [SbF_6_]^−^, [R_6_SO_4_]^−^, [OTs]^−^, [OMs]) that are used to demulsify, dehydrate, and desalt the crude oil [25]. Alkyl ammonium ionic liquids reduce the interfacial tension of the crude oil−water system and are used for enhanced oil recovery as an ionic liquid-polymer flooding technique [26]. The ILs and PILs are preferred for use as oilfield chemicals in the petroleum industry due to their high thermal stability, low vapor pressure, and their multi-functionalities as corrosion inhibitors. They have good interactions with petroleum crude oil constituents and have amphiphilic characteristics and high dispersion, as well as suspension and solubility in the different crude oil constituents. The literature survey indicated that no modification occurred to produce ILs or PILs from the asphaltenes or different crude oil constituents. In this respect, this work aims to modify the chemical structure of asphaltenes that are separated from crude oil, bitumen, and petroleum sludge to produce amphiphilic PILs. The precipitated asphaltenes were modified via oxidation of the lower aliphatic chain through carboxylation followed by conversion to asphaltene acid chloride that reacted with ethoxylated N-alkyl pyridinium derivatives PILs. Moreover, the carboxylation of asphaltenes was carried out using the Diels–Alder reaction with maleic anhydride that linked with ethoxylated N-alkyl pyridinium derivatives to produce amphiphilic asphaltene PILs. The modified new amphiphilic asphaltenes PILs were interacted with asphaltenes in the crude oil to replace the asphaltene layer on the emulsified water droplet for demulsification of the petroleum crude oil water emulsions. Moreover, there were structural variations of the new amphiphilic asphaltenes PILs used to prevent the aggregation and precipitation of asphaltenes of heavy and extra-heavy petroleum crude oils.

## 2. Experimental

### 2.1. Materials 

Heavy petroleum crude oils, their petroleum sludge, and bitumen (Ras Tanura oil, Aramco, Saudi Arabia) were used to separate the asphaltenes. The specifications of Arabic heavy crude oil (19.2° API) were summarized in Table 1. Toluene: n-heptane solvents (1:40 Vol. %) was used to isolate the asphaltene fractions from the bitumen and heavy crude oil. In this respect, a crude oil sample (0.5 g) was dissolved in toluene (1 mL), and n-heptane (40 mL) was added. The mixture was refluxed and allowed to stand for 4 h, then centrifuged at 5000 rpm for 30 min to separate the asphaltenes [27]. Asphaltenes were separated from the petroleum sludge of the heavy crude oil according to the previously reported method in Reference [28]. Petroleum sludge (50 g) was extracted using Soxhlet extraction with toluene (300 mL) followed by rotary evaporation to obtain toluene soluble organics (TSO). The remaining solid in the Soxhlet funnel (TIM) was re-extracted with 150 mL of tetrahydrofuran (THF) for 3 h followed by rotary evaporation with THF to obtain heavy asphaltene fraction (HAs). The TSO was solubilized in toluene followed by the addition of n-heptane (100 mL) to precipitate light asphaltene fractions that were filtered and dried for 2 h at 105 °C. The seawater with total dissolved salt (36,170 mg·L ^−1^) was obtained from the Arabian Gulf along the Saudi coast. 4-Aminopyridine (4-AP), tetraethylene glycol (TEG), tetradecyl bromide, dichlorodiethyl ether (DDE), 1-ethyl-3-(3-dimethylaminopropyl) carbodiimide (EDC), maleic anhydride (MA), hydrogen peroxide, KMnO_4_, concentrated sulfuric acid, and organic solvents were purchased from Sigma-Aldrich chemicals Co. (Missouri, MO, USA) and used as received. 

### 2.2. Techniques

#### 2.2.1. Preparation of Asphaltenes Carboxylic (ACA) and Acid Chlorides (As-COCl)

Asphaltenes flakes separated from bitumen, sludge, and crude oil (1.00 g,), as well as concentrated sulfuric acid (25 mL, >95% w/w) were mixed and stirred at 0 °C. KMnO_4_ (3 g) was added slowly under vigorous agitation while controlling the reaction temperature to never exceed 20 °C during mixing, followed by heating the reaction mixture to 80 °C for 30 min. Distilled water (50 mL) was then added while stirring for 15 min at 80 °C. The brown mixture was then diluted by the addition of 175 mL of water followed by dropwise addition of 10 mL of 30% v/v hydrogen peroxide. The yellow-green mixture was filtered, washed with 150 mL of 10% aqueous HCl and allowed to dry. The dry powder was dispersed in 200 mL of distilled water via ultra-sonication for 90 min. The dispersion was then centrifuged at 3000 rpm for 40 min and decanted to isolate the reaction products. The yield% of ACA was obtained as 95.8. The ACA (3g) was mixed with thionyl chloride (SOCl_2_, 100 mL) under stirring and refluxed for 12 h. The solids were separated by filtration and then washed with n-heptane several times and subsequently dried under vacuum at room temperature to obtain asphaltenes acid chloride (As-COCl) with a yield % of 83.5.

Asphaltenes/maleic anhydride adducts (AMA) was prepared by mixing asphaltenes (2 g) and maleic anhydride (MA; 1.5 g) in toluene (50 mL) in a three-necked bottom flask. The mixture was refluxed for 8 h under nitrogen atmosphere, followed by the removal of the solvent under reduced pressure. We washed the products with acetone to obtain an AMA with an 89.3 yield %. The AMA was hydrolyzed by refluxing with aqueous NaOH (10% W/V) for 3 h until the AMA suspension was solubilized completely. The AMA solution was cooled down, and the aqueous HCl (1M) was added to precipitate the AMA that separated. Then it was washed with dilute HCl (10 mM) and water and dried in a vacuum oven. 

#### 2.2.2. Preparation of Tetradecyl Pyridinium Bromide Ethoxylate 

The 4-AP (0.01 mol), DDE (0.01 mol), TEG (0.01 mol), and NaOH powder were mixed in the presence of xylene as solvent (100 mL) and then refluxed for 4 h from 100 to 180 °C. The solid powder separated by filtration and the xylene solvent evaporated under reduced pressure. The TEG was desalted using hot saline water, and the remaining organic layer was evaporated to obtain ethylated-4-amino pyridine (EAP) with a yield % of 95.3.

EAP (0.02 mol) was dissolved in dimethylformamide (DMF) (100 mL) and tetradecyl bromide (0.02 mol) was added dropwise to the solution under stirring at a reaction temperature of 150 °C for reaction times over 24 h. The DMF was evaporated, and the product was recrystallized from ethanol to obtain quaternized tetradecyl pyridinium bromide ethoxylate (QAP-Br) with a yield % of 68.3.

#### 2.2.3. Synthesis of Amphiphilic Asphaltenes Ionic Liquid Polymers 

As-COCl (2 mmol, 0.28 g) and QAP-Br (2 mmol) were dissolved in toluene (50 mL) in an ice bath temperature at −5 °C. Triethylamine (TEA, 2 mmol) was added to the reaction mixture, and the reaction temperature was kept at 25 °C for 24 h. The reaction product was separated after evaporation of the solvent and precipitations into heptane to obtain AIL with a yield % of 93.8. 

AMA (5 mmol, 1.06 g) and QAP-Br (5 mmol) were dissolved in chloroform (50 mL), and EDC (0.04 g) was added to the reaction mixture. The reaction mixture temperature was kept at 25 °C for 24 h. The reaction product was isolated after evaporation of the solvent and precipitation into heptane to obtain AIL-2 with a yield % of 78.3.

### 2.3. Characterization 

^1^HNMR spectra of the synthesized PILs were conducted using a 400 MHz Avance DRX-400 spectrometer (Bruker, Billerica, MA, USA). Surface tension and interfacial tension measurements were determined based on the pendant drop technique using a drop shape analyzer model DSA-100 (Kruss GmbH, Hamburg, Germany). Zeta potentials of the emulsion samples were determined using the Zetasizer Nano ZS; Malvern Instruments, Malvern, UK) at 25 °C. Dynamic light scattering (DLS; Zetasizer Nano) was used to investigate the particle size hydrodynamic diameter (nm) and polydispersity index (PDI) in an aqueous solution at 25 °C. An Olympus BX-51 microscope attached with a 100W mercury lamp was used to investigate the sizes of the dispersed phase droplets into the emulsions. The thermal stability and characteristics were evaluated using thermogravimetric analysis (TGA; Shimadzu DTG-60M, Tokyo, Japan) and conducted under a nitrogen atmosphere at a heating rate of 10 °C per minute. A differential scanning calorimeter (DSC; Mettler-Toledo, Kowloon, Hong Kong) equipped with a liquid nitrogen cooling accessory was used to investigate the thermal characteristics of the prepared PILs under the nitrogen purge (flow rate 60 mL/min). At least three runs occurred for all characterization procedures, following the same experimental protocol to ensure data reproducibility.

### 2.4. Application of Asphaltenes Ionic Liquids (As-ILs) as Demulsifier

Water-in-oil synthetic emulsions were prepared using freshly collected crude oil, free of demulsifier and seawater as the aqueous phase. All emulsions were prepared with a total volume of 50 mL. The ratio between crude oil and the aqueous phase was prepared by mixing the oil: seawater (volume:volume %) using a Silver stone homogenizer under continuous stirring (9000 rpm for 30 min) at room temperature to obtain a blank emulsion. Different concentrations of the prepared demulsifiers ranging from 100 to 5000 mg. L^−1^ were added after the emulsion preparation. The prepared PILs were dissolved in solvent xylene/ethanol (75/25 vol. %) with total concentrations of 50 wt. %. The synthesized PILs solutions were injected into the emulsion using a micropipette while using an oscillating shaker for 15 min. The bottle test was used to estimate the capability of the synthesized demulsifiers in breaking of water in oil emulsions at 65 °C using gravitational settling with graduated cylinders. The zero time was started after heating the cylinders in a water bath at 65 °C. The phase separation was recorded as a function of time during the settling process. The demulsification efficiency (DE%) can be calculated using the following Equation:

DE % = [ (V_o_ × 100)/(V_1_)], (1)

where V_o_ and V_1_ are volume (ml) of emulsified and separated water, respectively.

## 3. Results and Discussion

**Scheme 1:**
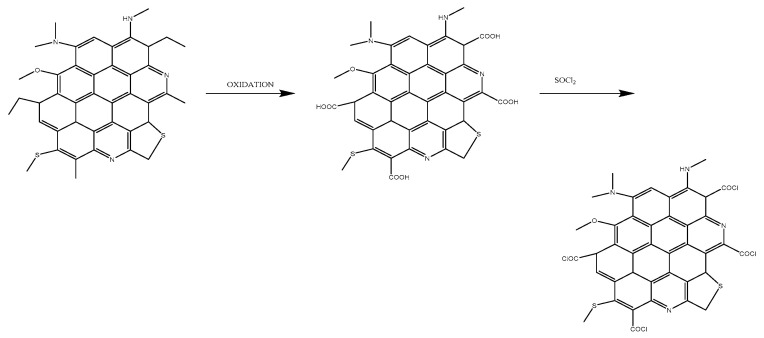
Synthesis of acid chloride (As-COCl).

**Scheme 2:**
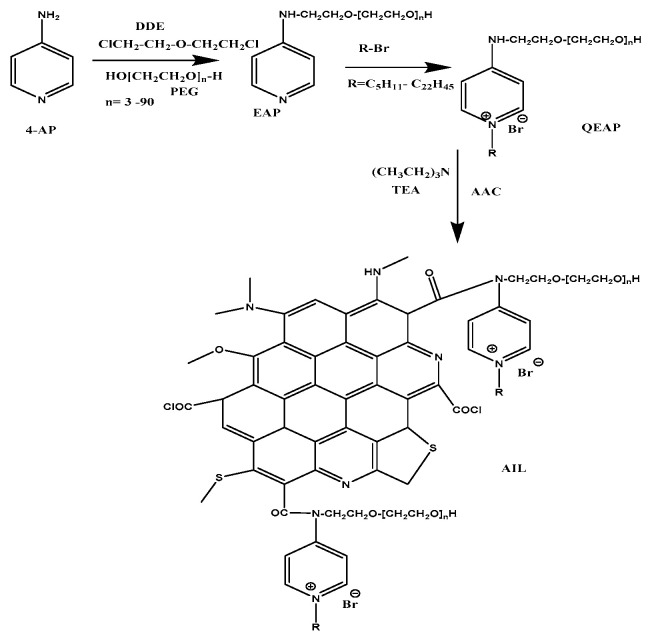
Synthesis of amphiphilic asphaltene ionic liquid (AIL).

**Scheme 3:**
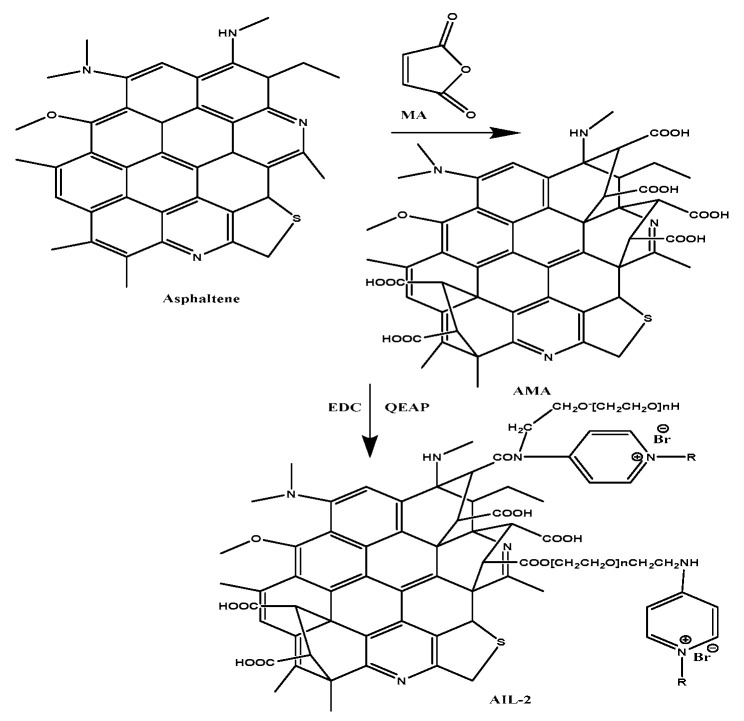
Synthesis of asphaltene IL (AIL-2).

Asphaltenes are defined as the fraction soluble in aromatic solvents such as toluene but insoluble in n-alkanes. They are the least soluble and the heaviest fraction in heavy oil. Asphaltene chemical structures generally contain polycondensed aromatics, heterocycles, and aliphatic alkyl groups, and their molecular weights are dependent on the origin, source, and types of crude oils [29]. The present work separated asphaltenes from Arabic heavy crude oil, and its asphalt, and petroleum sludge using toluene: heptane co-solvent as reported in the experimental section. The proposed simple model of asphaltenes separated from Arabic heavy crude oils, and their products are represented in Scheme 1 [30]. The present work aims to modify the asphaltene chemical structure to synthesize new amphiphilic asphaltenes ILs by oxidation of the alkyl chain on the periphery of asphaltenes to the carboxylic group ACA. This was followed by conversion to acid chloride (As-COCl) by reacting with SOCl_2_ as represented in Scheme 1. The As-COCl was converted to amphiphilic asphaltene ionic liquid (AIL) by reacting with QAP-Br using trimethylamine as a catalyst as clarified in Scheme 2. The R group in the chemical structure of QAP-Br was the tetradecyl group that was selected among alkyl groups that affect the polarity and amphiphilicity of pyridinium ILs [31,32]. The second type of asphaltene IL (AIL-2) was prepared after reacting asphaltenes with MA, followed by esterification or amidation with QAP-Br in the presence of EDC as represented in Scheme 3 [32]. The anhydride group was converted to a carboxylic acid group during the purification, and it was converted to amide with EDC as reported previously in Reference [32].

### 3.1. Characterization of Amphiphilic Asphaltene ILs

**Figure 1:**
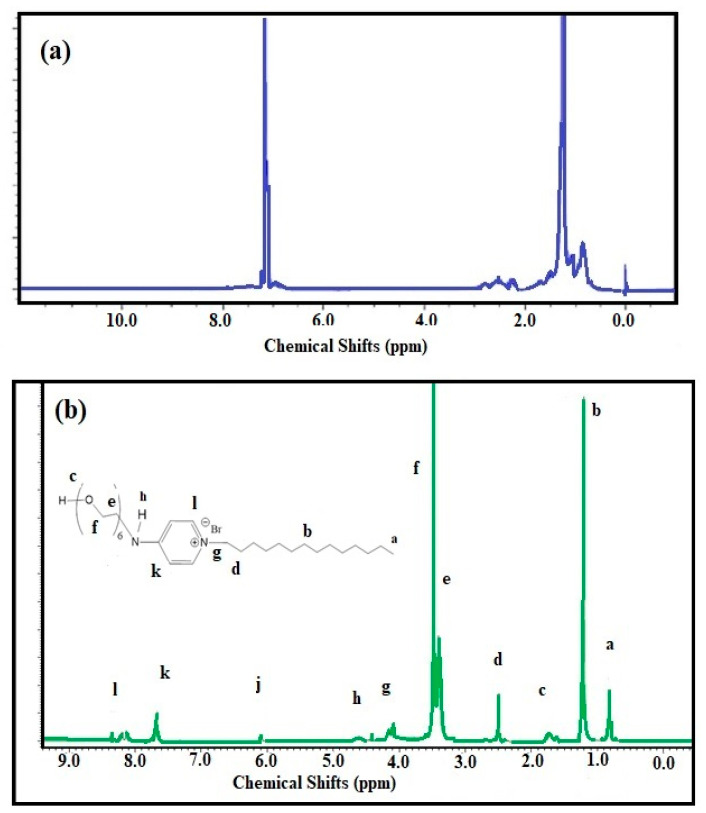
^1^HNMR spectra of (**a**) asphaltenes and (**b**) QAP-Br.

**Figure 2:**
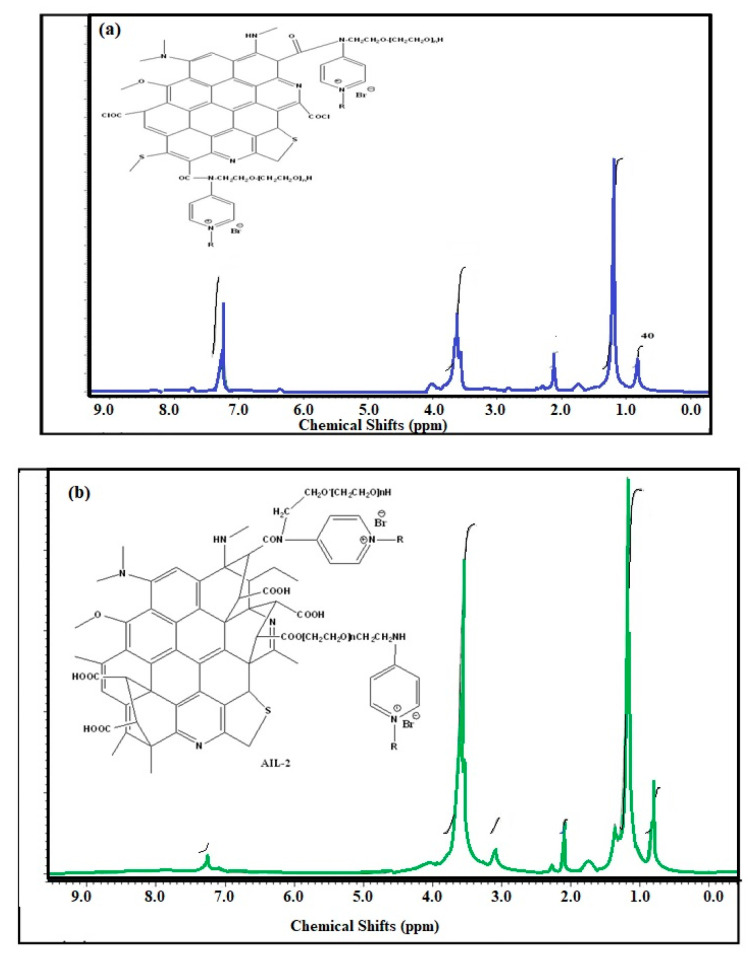
^1^HNMR spectra of (**a**) AIL and (**b**) AIL-2.

The chemical structures of the asphaltenes, ACA, As-COCl, and AMA were elucidated from their FTIR spectra represented in the Supplementary Information (Figure S1a–d). The lower acid number of heavy crude oil (Table 1) elucidated the lower naphthenic acid and carboxylic acid contents of its asphaltenes. The FTIR spectra of asphaltenes (Figure S1a) confirmed the presence of aliphatic and aromatic hydrocarbons from the appearance of bands at around 2980 and 2750 cm^−1^ assigned to the stretching of the C–H aliphatic and at 3150–300 cm^−1^ or weak band at 1600 cm^−1^, referred to as stretching of the C–H or C=C aromatics, respectively. The new bands at 3500 and 1710 cm^−1^ for OH and C=O stretching in the spectra of the ACA and AMA (Figure S1 b,d) confirmed the carboxylation of asphaltenes via oxidation of alkyl groups to produce ACA. The MA ring opening followed the Diels–Alder reaction with MA. The lower intensity of bands at around 2980 and 2750 cm^−1^ assigned to the stretching of the C–H aliphatic of asphaltenes (Figure S1a) in spectra of ACA (Figure S1b) and As-COCl (Figure S1c) show the oxidation of aliphatic alkyl groups of asphaltenes. A new band at 1780 cm^−1^ in the As-COCl spectrum (Figure S1c) confirmed the conversion of the carboxylic group of ACA to its acid chloride via a reaction with SOCl_2_ (Scheme 1). The FTIR spectra of QEP-Br ethoxylate and their asphaltenes ionic liquid polymers (AIL and AIL-2) are summarized in Figure S2a–c. The bands at 3316–3289, 3117–2780, 1656, 1543, 1182, 980, and 830 cm^−1^ were referred to as OH stretching, CH aromatic and aliphatic stretching, C=C stretching, C–NH bending, C–O stretching, (CH_2_)_n_ bending, and C=C bending, respectively. These bands confirmed the ethoxylation and quaternization of 4-AP to form EAP (Scheme 2). The appearance of new bands at 1690 and 1720 cm^−1^ represented C=O stretching (Figure S2b–c) confirmed the amidation of As-COCl and AMA and the formation of AIL and AIL-2. The chemical structure of the asphaltenes and QAP-Br was shown from the ^1^HNMR spectra as seen in Figure 1a,b. The ^1^HNMR spectra of AIL and AIL-2 are summarized in Figure 2a,b. The chemical structure of asphaltenes could be confirmed from its ^1^HNMR spectrum (Figure 1a) to show that the asphaltenes contained a significant quantity of paraffinic H atoms (CH_3_, CH_2_), a relatively low amount of mono aromatic and polyaromatic H atoms (5%), and a negligible percentage of olefins [30]. The chemical structure of QAP-Br was marked and clarified in its ^1^HNMR spectrum (Figure 1b). The appearance of peaks at 8.3, 6.1, 4.6, 3.5, and 0.84 ppm was referred to as aromatic (H), ^+^N–CH_2_, ^+^N–C–CH_2_, OCH_2_CH_2_–, and CH_3_ protons of QAP-Br [32]. The disappearance of the broad peak at 4.6 ppm related to NH (Figure 1b) and appearance of a strong peak at 7.24 ppm in the AIL spectrum (Figure 2a) confirmed the formation of amide groups between QAP-Br and As-COCl to produce AIL. ^1^HNMR spectrum of AIL (Figure 2a) showed that the integration ratios of peaks appeared at 0.8 and 1.2 ppm, and were related to (CH_2_)_n_ and –CH_3_ of the alkyl chain of QAP-Br. There were no additional –CH_3_ or –CH_2_ groups added from As-COCl. This meant that all aliphatic alkyl groups of asphaltene (Scheme 1) converted to acid and acid chloride. Moreover, the high integration of the peak at 7.2 ppm of AIL and AIL-2 (Figure 2a,b) compared to that determined in the ^1^HNMR spectrum of QAP-Br (Figure 1b) confirmed the incorporation of polycondensed aromatics of asphaltene in the chemical structure of AIL and AIL-2. The proposed chemical structure of AIL-2 represented in Scheme 3 is confirmed from its ^1^HNMR spectrum represented in Figure 2b. The intensity of peaks at 1.2 ppm (S, CH_2_ aliphatic protons), and 7.26 ppm (S, aromatic protons) in the AIL-2 spectrum (Figure 2b) showed the decreasing of aromatic hydrocarbon intensity more than aliphatic intensity to confirm that the reaction of QAP-Br with AMA occurred. The increasing of –OCH_2_–CH_2_– integration in the spectrum of AIL-2 (Figure 2b) more than AIL (Figure 2a) confirmed the higher reactivity of QAP-Br towards AMA to form amide or ester groups (Scheme 3). The appearance of peaks at 3.09 ppm in the spectrum of AIL-2 (Figure 2b) proved the esterification of QAP-Br hydroxyl groups with anhydride groups of AMA. The ^13^CNMR spectrum of AIL is shown in Figure S3 to confirm the asphaltenes aromatics, CH_3_, CH_2_ of tetradecyl, and CH_2_O of ethoxylated. This was via the appearance of –COCl and –CONH groups as marked on the peaks of AIL.

**Figure 3:**
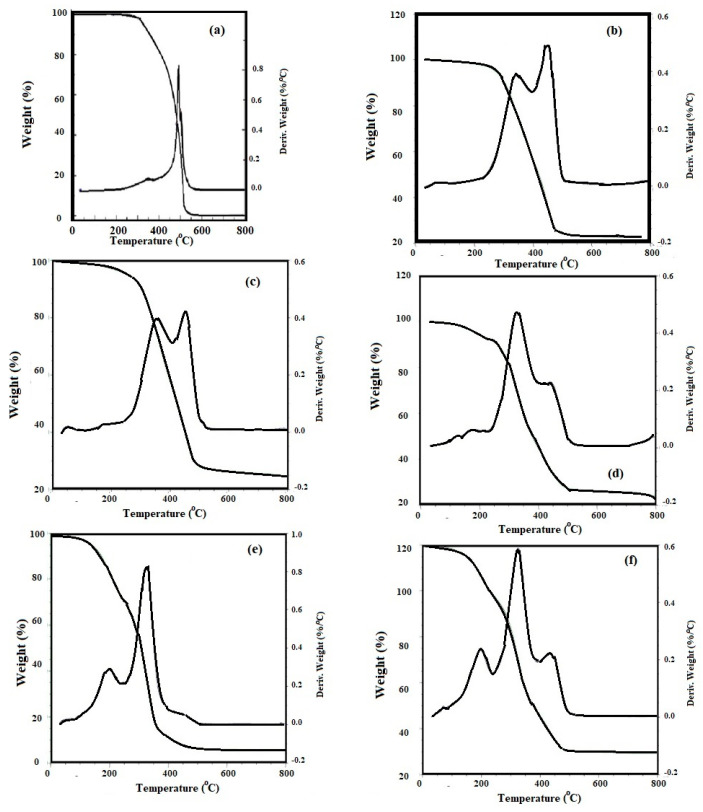
TGA and DTA thermograms of (**a**) asphaltenes, (**b**) AMA, (**c**) ACA, (**d**) QAP-Br, (**e**) AIL, and (**f**) AIL-2.

The thermal stability of PILs is one of the important parameters that justify their preferred application in the petroleum field. There is no need to expand its operations at an elevated temperature. The data of thermogravimetric and differential thermogravimetric (TGA-DTG) thermograms of asphaltenes, AMA, ACA, QAP- Br, AIL, and AIL-2 are shown in Figure 3a–f. The data show that the thermal stability of asphaltene (Figure 3a) increased after modification to AMA (Figure 3b) and ACA (Figure 3c). The initial degradation temperatures, IDT, of asphaltenes, AMA, and ACA were 173 °C, 250 °C, and 290 °C, respectively. Moreover, there was a drastic decomposition of native at temperatures ranging from 367 to 600 °C. The weight loss % of asphaltenes (75.5 wt. %), AMA (65 wt. %), and ACA (60 wt. %) was accompanied by the appearance of endothermic and exothermic effects and the elimination of water (H_2_O) and carbon dioxide (CO_2_). The remaining residues of AMA and ACA above 650 °C were transformed into coke due to the formation of cyclic derivatives (pyrolysis: elimination of nitrogen N_2_, ethylene C_2_H_4_, and ethyl CH_3_–CH_2_–) [33]. We also noticed that the thermal stability of AIL (Figure 3e) and AIL-2 (Figure 3f) showed new additional peaks at 450 °C, which also appeared in the QAE-Br thermogram (Figure 3d) to confirm the reaction of As-COCl or AMA with QAE-Br. The AIL (Figure 3e) and AIL-2 (Figure 3f) thermograms contain three to five temperature transitions, namely, the elimination of water molecules adsorbed on their surfaces and in the bulk of native asphaltenes, As-COCl or AMA, and QAE-Br; the section corresponding to the evolution of carbon dioxide gases; and the section of the transformation of AIL (Figure 3e) and AIL-2 (Figure 3f) in coke. Esterification or amidation of As-COCl or AMA with QAE-Br increases the thermal stability of QAE-Br, which is referred to as the formation of new strong chemical bonds and replacement of hydrogen by new modified asphaltene functional groups [34]. The AIL (Figure 3e) and AIL-2 (Figure 3f) thermograms indicated that their weight losses were completed in three steps. The sample kept thermostable below 200 °C. It began to lose its weight at about 210 °C, reaching the maximum rate of weight loss at 320 °C, and it completely lost its weight when the temperature reached 430 °C which was similar to alkyl pyridinium bromide ILs [35]. 

**Figure 4:**
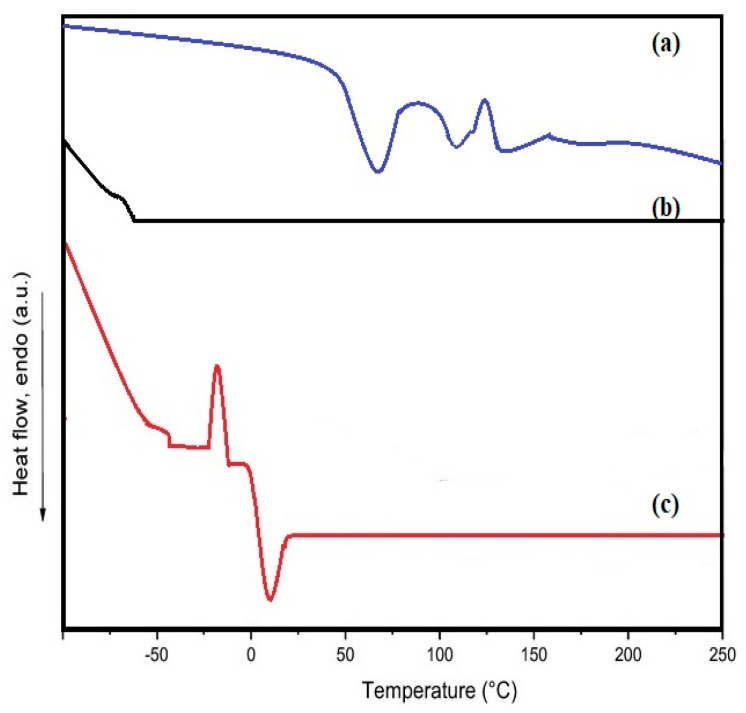
Differential scanning calorimeter (DSC) thermograms of (**a**) asphaltenes, (**b**) AIL and (**c**) AIL-2.

The thermal characteristics of asphaltenes and their amphiphilic PILs (AIL and AIL-2) were evaluated from their DSC thermograms, as shown in Figure 4a–c. The DSC thermograms of asphaltenes (Figure 4a) shows no clear baseline that referred to their complex chemical nature [36]. There was a broad endothermic peak at 60 °C that could be identified, which attributed to the amorphous mesophase [36]. The modified structure of asphaltenes AIL and AIL-2 thermograms (Figure 4b,c) exhibited different thermal characteristics involving the glass transition temperature (T_g_) at −68 °C and −45 °C, respectively. It was noticeable that the Tg value of AIL-2 increased slightly compared to AIL to show that AIL-2 was more ridged than AIL due to amidation and esterification of AMA more than As-COCl with QAP-Br [37]. The presence of COOH in the chemical structure of AIL-2 exhibited large ligand field stabilization energies that enhanced the T_g_ values [37]. Furthermore, the increase in T_g_ can be correlated to a decrease in free volume that is caused by a distorted packing of the macromolecular chains of AIL-2 [38]. These interactions affected the thermal characteristics of AIL-2 with the appearance of characteristic temperatures involving the T_g_, crystallization temperature (T_c_), and the melting point (T_m_), at −45 °C, −20 °C, and 7.8 °C, respectively. The lowering T_g_ and T_m_ values of AIL and AIL-2 proves their tendency to be an ionic liquid [39]. 

### 3.2. Surface Activity of Amphiphilic Asphaltene PIL

**Figure 5:**
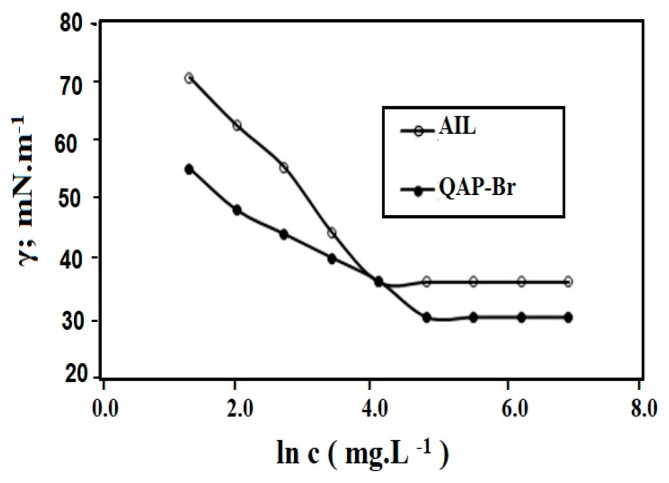
The relation between surface tension and different concentrations of QAP-Br. and AIL at 25 °C.

**Figure 6:**
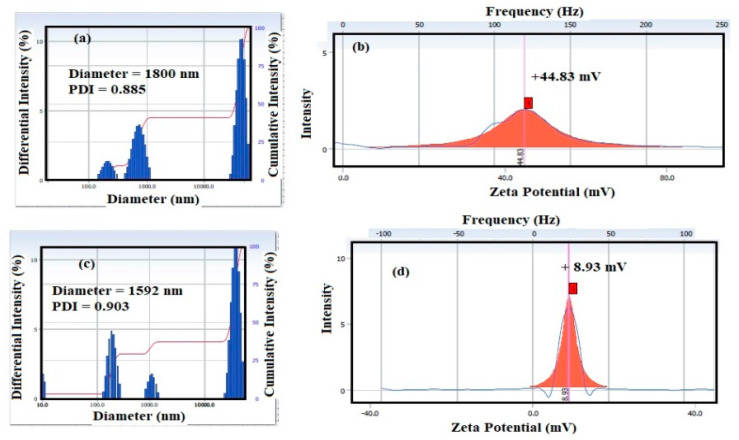
Dynamic light scattering (DLS) and zeta potential data of (**a**), (**b**) AIL, (**c**) and (**d**) QAP-Br in aqueous solution.

Most of the chemicals used in the petroleum oilfield industries should possess amphiphilic and surface activity due to the presence of both hydrophobic and hydrophilic constituents in the chemical composition of crude oils [40]. The surface activity of the prepared QAP-Br, AIL, and AIL-2 at the water–air interface was investigated from the surface tension measurement. It is expected that the presence of different active sites in the chemical structure of QAP-Br, AIL, and AIL-2 affects their performance in their aqueous solutions. It was noticed that the AIL-2 did not become soluble in water, but it was soluble in alcohol. This can be referred to as the esterification of hydroxyl groups of QAP-Br with AMA (Scheme 3), which could reduce the hydrophilicity of AIL-2. The solubility of QAP-Br, AIL, and AIL-2 in water can be estimated from the relative solubility number (RSN). It was measured as the volume of water (mL) used to obtain a turbid solution of the prepared materials (1 g) in 30 mL solution consisting of 96 wt.% dioxane and 4 wt.% toluene. The RSN values of the prepared EPA, AIL, and AIL-2 were determined and are listed in Table 2. It was previously reported that the materials having an RSN value more than 17 and a lower area are more water-soluble, and more oil-soluble, respectively [41]. The RSN value of QAP-Br and AIL (Table 2) indicated that they could be solubilized in both polar and nonpolar organic solvents. The RSN value of AIL-2 confirmed its insolubility in water. This meant that the esterification of the hydroxyl groups of TEG reduced the water solubility of AIL-2 in water due to the hydrophobic interactions of asphaltene phenyl groups and alkyl group of QAP-Br. The relation between surface tension (γ; mN·m^−1^) measurements of QAP-Br and AIL versus their concentrations (ln c, mg·L^−1^) in aqueous solutions in the water at 25 °C is shown in Figure 5. The critical micelle concentrations (cmc; mg·L^−1^) are determined from Figure 5, and it is measured at the concentration where γ starts to increase. The corresponding surface tension at cmc (γ_cmc_) is tabulated in Table 2. The data listed in Table 2 confirm that the solubility of QAP-Br in water is reduced by the reaction with the hydrophobic moieties of asphaltene to produce AIL. This is determined from the lowering cmc of QAP-BR from 125 to 62.5 mg·L^−1^ of AIL. Moreover, the surface tensions at the cmc (γcmc; Table 2) data proved that the water surface tension of QAP-Br was reduced more than AIL. It seems reasonable to propose that the AIL has hydrophobic groups of asphaltene unfolding its tails and loops to cover the entire interface, and thus, oriented and packed AIL adsorption at the air–water interface. The DLS measurements, including both aggregation diameters and surface charges (zeta potentials; mV) for QAP-Br and AIL in water, were used to confirm the surface tension measurements as shown in Figure 6,a–d. The data confirmed that the aggregation diameter of the AIL (Figure 6a) and its surface charges (Figure 6b) was 1800 nm and 44.83 mV, which changed for QAP-Br (Figure 6c,d) to 1592 nm and +8.93 mV, respectively. These data agree with the cmc data to confirm that the presence of asphaltene in the chemical structure of AIL increases the hydrophobic interaction of QAP-Brand increases the micelle diameter (aggregation) and their positive charges due to presence of pyridinium cations [42]. The effectiveness of QAP-Br and AIL to reduce the surface tension (π_cmc_; mN·m^−1^) was calculated from the following Equation:

π_cmc_ = γ_o_ − γ_cmc,_ (2)

where γ_o_ is the water surface tension (72.1 mN·m^−1^). The higher π_cmc_ value of QAP-Br more than AIL (Table 2) indicated that the QAP-Br interacted with water via a dipole–dipole interaction mechanism more than AIL [43,44]. 

The adsorption of QAP-Br and AIL molecules at the air–water interface was used to evaluate their surface activities. The concentration of QAP-Br and AIL molecules adsorbed per unit area of the interface was designated as the surface excess concentration (Γ_max_). It can be calculated from Equation (3):

Γ_max_ = [(−δγ/δ ln c)_T_/(RT)], (3)

where R and T are constant equals 8.314 J mol^−1^ K^−1^ and temperature (K) of measurements, respectively. The relation between A_min_ and Γ_max_ is shown in Equation (4):

A_min_ = [(10^16^)/(NΓ_max_)], (4)

where N is Avogadro’s number is used to calculate A_min,_ which is summarized in Table 2. The A_min_ was used to determine the orientation and packing degrees of the adsorbed QAP-Br and AIL molecules, at the interfaces. The increasing of the Γ_max_ value for AIL than QAP-Br (Table 2) indicates the higher adsorption of AIL at the air–water interface, which also reflects a reduction of the water surface tension. The increasing of the Γ_max_ and lowering A_min_ values could be explained on the basis that the interactions between the hydrophilic arms of the AIL molecules increased the packing of molecules at the interfaces [44]. The lower A_min_ (0.033 nm^2^/molecule) of AIL suggested that its adsorption oriented away from the liquid in a more tilted position. However, the complete surface coverage of AIL more than QAP-Br at the air–water interface was confirmed from the low A_min_ and high Γ_max_ values.

### 3.3. Application of the Prepared AIL and AIL-2 as Demulsifier for Petroleum Crude Oil Emulsions

The ability of chemical demulsifiers to dehydrate the water petroleum crude oil emulsions is based on their quick diffusion into the continuous phase of the emulsion and fast adsorption on the dispersed phase at the water–oil interface [45]. The replacement of the asphaltene layer on the surface of the dispersed droplet probably occurred from the reduction of the interfacial property of emulsion followed by droplet coalescence and sedimentation. The replacement of the asphaltene rigid films at interfaces with the formation of soft film and reduction of the interfacial tension are essential parameters. The present works aimed to use asphaltene modified PIL mixtures based on AIL and AIL-2 in the presence of QAP-Br as chemical demulsifiers to replace the asphaltenes at the oil–water interfaces. The demulsifier compositions of the AIL, AIL-2, and QAP-Br blends were coded as M1 to M7 and are summarized in Table 3. The xylene/ethanol 75/25 (vol. %) solvent was used to solubilize the demulsifier blends. The interfacial tension (IFT; mN·m^−1^) between crude oil emulsions O/W and M1-M7 water solutions were determined and listed in Table 3. The type of synthetic crude oil water emulsions ranged from 90:10 to 50:50 and is evaluated using the drop test method to confirm that two emulsions are dispersed in toluene to show the formation of water-in-oil (W/O) emulsions. The 10:90 crude oil: water emulsion is an O/W type as dispersed in water. The data of the IFT shown in Table 3 reflects that 1000 ppm (mg·L^−1^) of M1-M3 aqueous solutions were reduced with increases in the water contents in emulsions. Moreover, the reduction of IFT varied with the demulsifier compositions from M4 to M7. The M7 demulsifier (contains high QAP-Br content) showed a greater reduction in IFT with the increasing of water contents of the emulsion (O:W; 10:90). M6 and M4 showed a lowering in the IFT for the O:W emulsion (90:10). M5 showed a greater reduction in the IFT values using the W:O (50:50). These data showed the ability of the demulsifier blends to penetrate into the rigid asphaltene layer on the dispersed water or oil droplets to demulsify the dispersed phase [46]. The great reduction of IFT values in short times from 5 to 20 min showed the formation of mixed interfacial films with asphaltene at the oil–water or water–oil interfaces.

**Figure 7:**
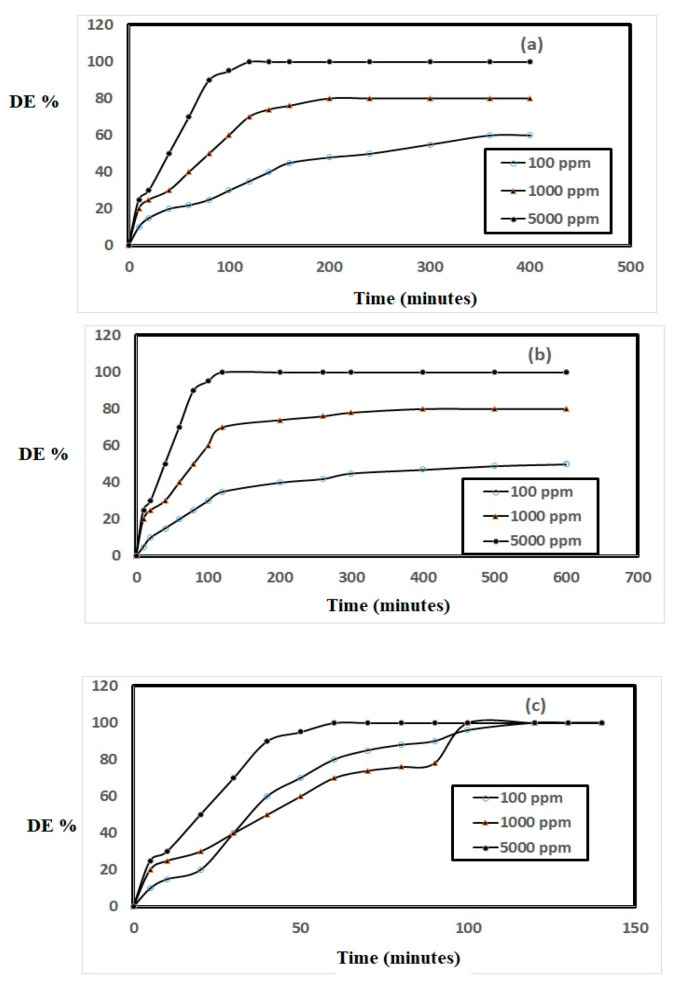
Relation of DE% and demulsification times of different concentrations of (**a**) M5 with emulsion 50:50, (**b**) M6 with emulsion 90:10, and (**c**) M7 with emulsion 10:90 (O:W) at 65 °C.

**Figure 8:**
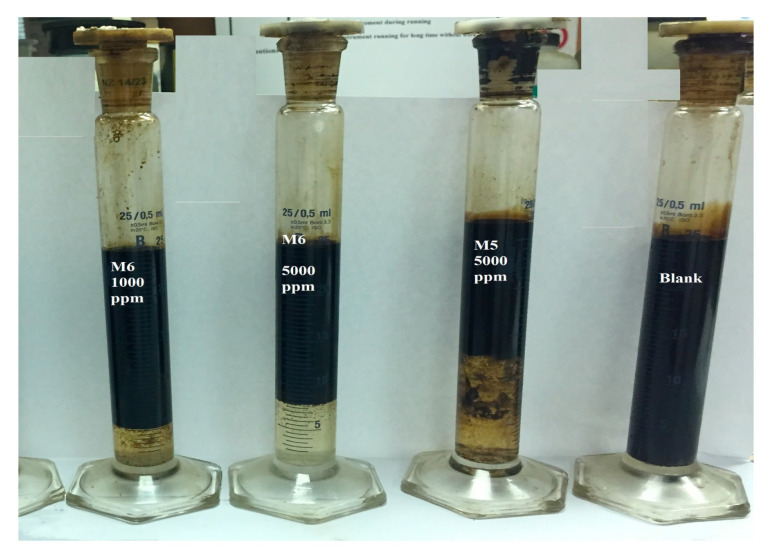
Demulsification photos of petroleum crude oil water emulsions in the presence of M5 and M6 and absence of demulsifier (blank).

The blends M5 to M7 showed greater IFT reduction with stable equilibrium values during short times ranging from 5 to 20 min and were selected as emulsifiers for crude oil water emulsions. Different concentrations of M5-M7 blends ranging from 100 to 5000 ppm were used to demulsify the different crude oil emulsions using a conventional heating method, as described in the experimental section. The DE (%) and the separation settling times were determined and listed in Table 4. The relations between the DE (%) and the separation time of crude oil emulsions using different concentrations of M5-M7 are shown in Figure 7. The photos of water separations of crude oil emulsions using M5-M7 were clarified in Figure 8. Careful inspection of the data listed in Table 3 and Figure 7a−c indicate that the DE (%) 100 occurred in the presence of 5000 ppm of M5, M6, and M7 for the O:W emulsions 50:50, 90:10, and 10:90, respectively. The demulsification times of M5, M6, and M7 for the O:W emulsions 50:50, 90:10, and 10:90, were 120, 120 and 60 min, respectively. Accordingly, the ability of M5-M7 to replace asphaltenes layers at emulsion droplets controls the DE (%) data was affected by the demulsifier compositions. The kinetics dehydration curves (Figure 7a–c) of the crude oil emulsions showed s-curves to clarify the demulsification mechanism of crude oil emulsions. The first stage confirmed the diffusion of demulsifiers, the second stage proved the replacement of asphaltenes films, the third stage represented the collection of the dispersed droplets either water or oil, and the last fourth stage clarified the sedimentation process [47]. The first step of the s-curves (Figure 7a–c) was slow and showed that the diffusion of M5-M7 demulsifiers in the crude oil emulsions controlled the rate of dehydration and mechanism of the demulsification process [47].

**Figure 9:**
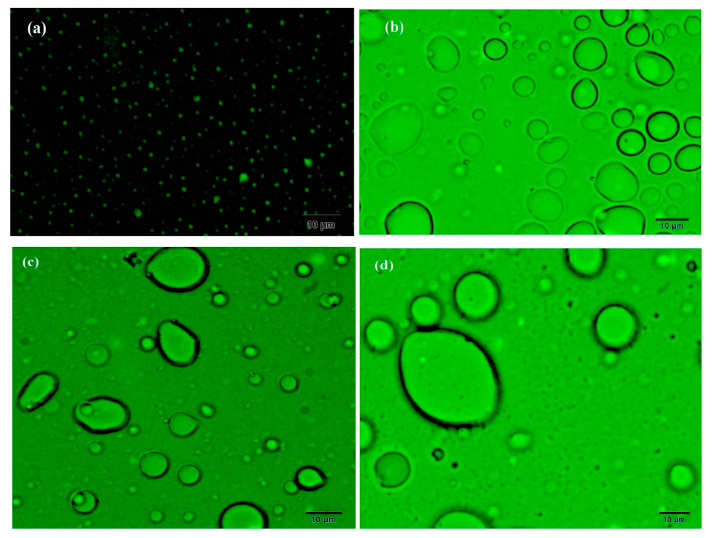
Optical microscopic photo of crude oil:water emulsion (50:50) (**a**) blank after 7 days, (**b**) after injection with 100 ppm of M5, (**c**) after injection with 1000 ppm of M5, and (**d**) after injection with 5000 ppm of M5.

The effects of M5 demulsifier concentrations on the sizes of a dispersed water droplet was evaluated using an optical microscope and represented in Figure 9a–d. We noticed that the droplet sizes of the emulsions increased with increases in the M5 concentrations than the blank emulsion (Figure 9a). We also observed that the films surrounding the dispersed droplets was partially destroyed due to the strong bridging interaction and charging neutralization between the AIL, AIL-2, and QAP-Br cations and the anion with the heteroatoms of asphaltene macromolecules. The protective film surrounding the water droplet was replaced with AIL or AIL-2, and the thickness of this layer was reduced with increases in the size of a water droplet (Figure 9d). At this point, the asphaltene layers started moving away from the water droplet surface, and more AIL or AIL-2 interacted with the water droplets. The new AIL or AIL-2 interfacial films provided an excellent site for the aggregation of the small water droplets. The dipole–dipole interaction and hydrogen bonds between the hydrophilic moieties of AIL or AIL-2 made the water droplets close contact, and the water droplets finally coalesced to form big droplets to separate using gravity force from crude oil-water emulsions. The ability of AIL or AIL-2 to aggregate the asphaltene to small sizes will generate more voids on the water droplet, making it easier for the AIL or AIL-2 macromolecule to interact with the water droplet surface, thereby facilitating the demulsification process. 

## 4. Conclusions

New amphiphilic asphaltene ionic liquid polymers were prepared to obtain flexible chain PILs, which succeeded in reducing the water surface tension and water crude oil interfacial tension. The lower A_min_ (0.033 nm^2^/molecule) of AIL suggests that its adsorption oriented away from the liquid in a more tilted position. The significant reduction of the IFT in the presence of asphaltene ionic liquid polymers below 1 mN·m^−1^ in short times from 5 to 20 min represents the formation of mixed interfacial film with asphaltene at the oil–water or water–oil interfaces. Different blends of the prepared asphaltene PILs were demulsified at different compositions of the petroleum crude oil-water emulsion. The amphiphilic asphaltene ionic liquid interacts with asphaltene layers at the surface of the water droplets, thereby destabilizing the water crude oil emulsions.

## Figures and Tables

**Table 1:** Arabic heavy crude specifications.

Test	Method	Result
API gravity (degree)	ASTM-D5002	19.2°
Specific gravity 60/60 (°F)	ASTMD-1298	0.939
Wax content (wt. %)	UOP 46/64	5
Asphaltenes content (wt.%)	IP 143/84	13
Pour point (°C)	IP 15/67(86)	18
Salinity (NaCl; wt. %)	ASTM-D3230	0.24
Water content (wt. %)	ASTM D1744	0.145
Total acidity (mg of KOH/g of oil)	ASTM D664	2.353

**Table 2:** Surface activity parameters of QAP-Be and AILin water at 25 °C.

Polymers	cmc (mg·L^−1^)	γ_cmc_ mN·m^−1^	π_cac_ mN·m^−1^	−∂ γ/∂ ln c	Γ_max_ × 10^10^ mol/cm^2^	A_min_ nm^2^/molecule	RSN (mL)
QAP-Br	125 ± 0.3	30.2 ± 0.2	42	6.75	2.73	0.060	14.8
AIL	62.5 ± 0.5	36.2 ± 0.4	36	12.38	5.01	0.033	13.5
AIL-2	-	-	-	-	-	-	10.3

**Table 3:** Interfacial tension (IFT) values of different blends of QAP-Br, AIL and AIL-2 aqueous solutions versus water crude oil emulsions.

Code	Demulsifier Composition Wt. %			IFT (mN.m^−1^) Crude Oil: Water		
	AIL	AIL-2	QAP-Br	90:10	50:50	10:90
M1	100	0	0	2.5	1.3	0.83
M2	0	100	0	13.3	15.8	20.3
M3	0	0	100	1.3	0.53	0.13
M4	60	20	20	0.23	1.35	3.23
M5	40	20	40	1.34	0.55	1.23
M6	30	20	50	0.53	1.45	3.53
M7	20	20	60	2.31	1.23	0.88

**Table 4:** Demulsification efficiencies and their times at different concentrations of M5-M7 for different crude oil emulsions at 65 °C.

Demulsifier Blends	Conc. (ppm)	Demulsification Data					
		90:10		50:50		10:90	
		DE%	Time (min)	DE%	Time (min)	DE%	Time (min)
M6	100	50	600	20	600	10	600
	1000	80	400	30	500	20	480
	5000	100	120	70	360	40	360
M5	100	60	400	60	360	40	320
	1000	70	360	80	240	50	280
	5000	80	340	100	120	70	240
M7	100	25	500	40	400	100	120
	1000	30	450	60	350	100	100
	5000	40	400	70	300	100	60

## Supplementary Materials

The following are available online at https://www.mdpi.com/2073-4360/12/6/1273/s1, Figure S1. FTIR spectra of (a) asphaltene (b) ACA, (c) As-COCl, (d) AMA, Figure S2. FTIR spectra of (a) QAP-Br ethoxylate, (b) AIL and (c) AIL-2, Figure S3. 13CNMR spectrum of AIL.
