# Supplementary material for: Synthesis and Application of New Amphiphilic Asphaltene Ionic Liquid Polymers to Demulsify Arabic Heavy Petroleum Crude Oil Emulsions
Source: Polymers (Basel). 2020 Jun 2;12(6):1273. doi: 10.3390/polym12061273 (PMC7362221; doi:10.3390/polym12061273)
Supplement: Supplementary file 1 [file polymers-12-01273-s001.pdf]

Supporting information file

for

Synthesis and Application of New Amphiphilic Asphaltene Ionic Liquid Polymers to Demulsify Arabic Heavy Petroleum Crude Oil Emulsions

Ali I. Ismail[1*], Ayman M. Atta[2*], Mohamed El-Newehy[2,3], and Mohamed E. EL-Hefnawy[1,3]

[1]Department of Chemistry, Rabigh College of Arts and Sciences, King Abdulaziz University, Jeddah, Saudi Arabia (E-mail: A.I.I. aeyesmaeel@kau.edu.sa; M.E.E. malhefnawy@kau.edu.sa).

[2]Department of Chemistry, College of Science, King Saud University, Riyadh 11451, Saudi Arabia (E-mail: A.M.A. atta@ksu.edu.sa; M.E. melnewehy@ksu.edu.sa).

[3] Department of Chemistry, Faculty of Science, Tanta University, Tanta 31527, Egypt.

Crosspondance Email: AMA, atta@ksu.edu.sa; AII, aeyesmaeel@kau.edu.sa

The chemical structures of asphaltenes modification were elucidated using Fourier transform infrared analysis (Nicolet Magna 750 FTIR spectrometer using KBr, Newport, NJ, USA) in KBr.

The ^{1}HNMR and ^{13}CNMR spectra evaluated by using a 400 MHz Avance DRX-400 spectrometer (Bruker, Billerica, USA) in $CDCl_3$ solvent at 25 °C.

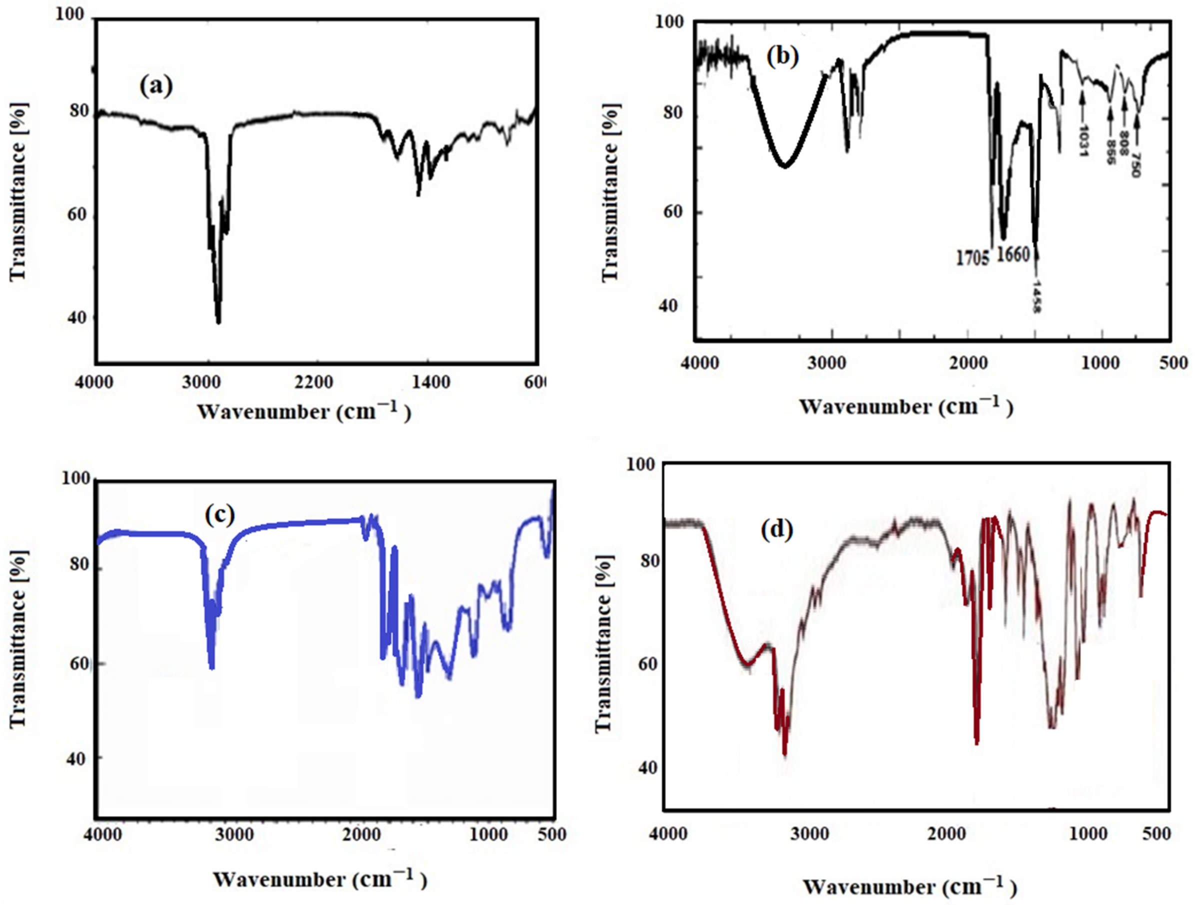

Fig. S1. FTIR spectra of a) asphaltene b) ACA, c) As-COCl, d) AMA.

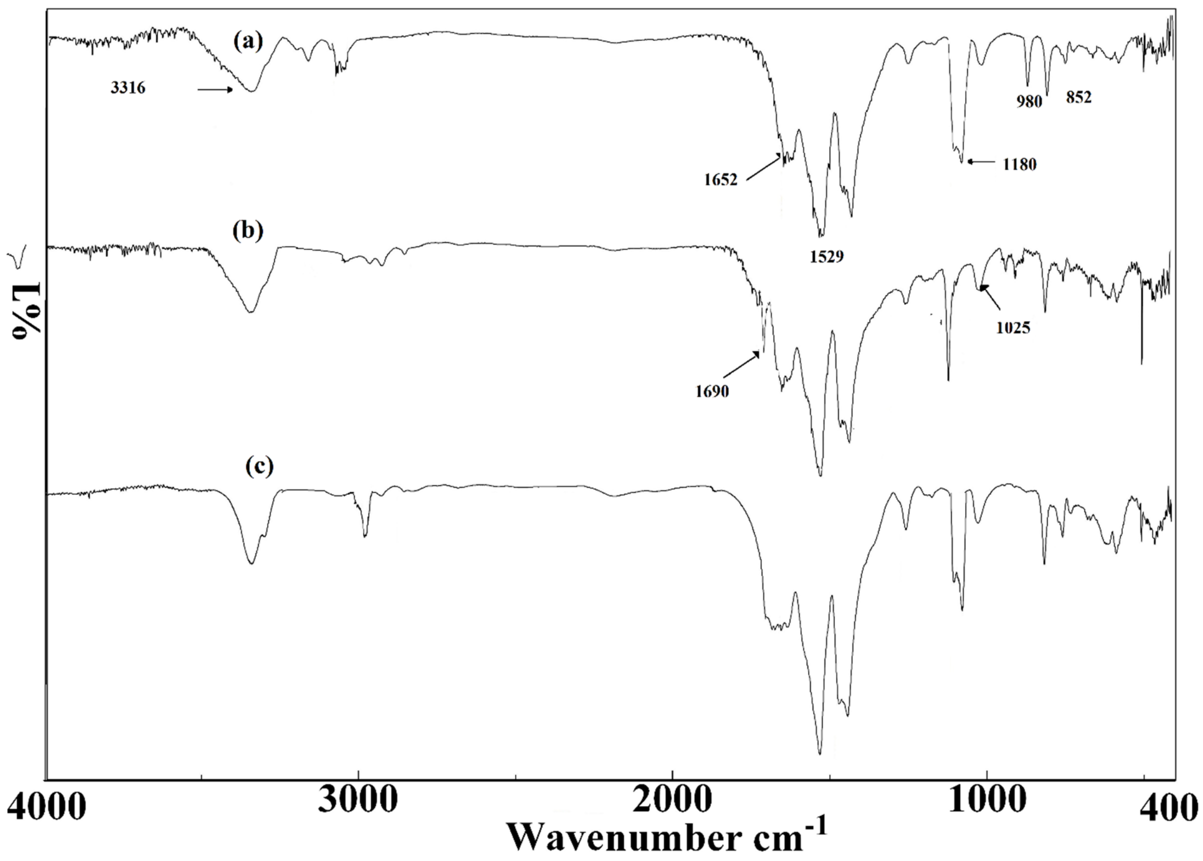

Fig. S2. FTIR spectra of a) QAP-Br ethoxylate, b) AIL and c) AIL-2.

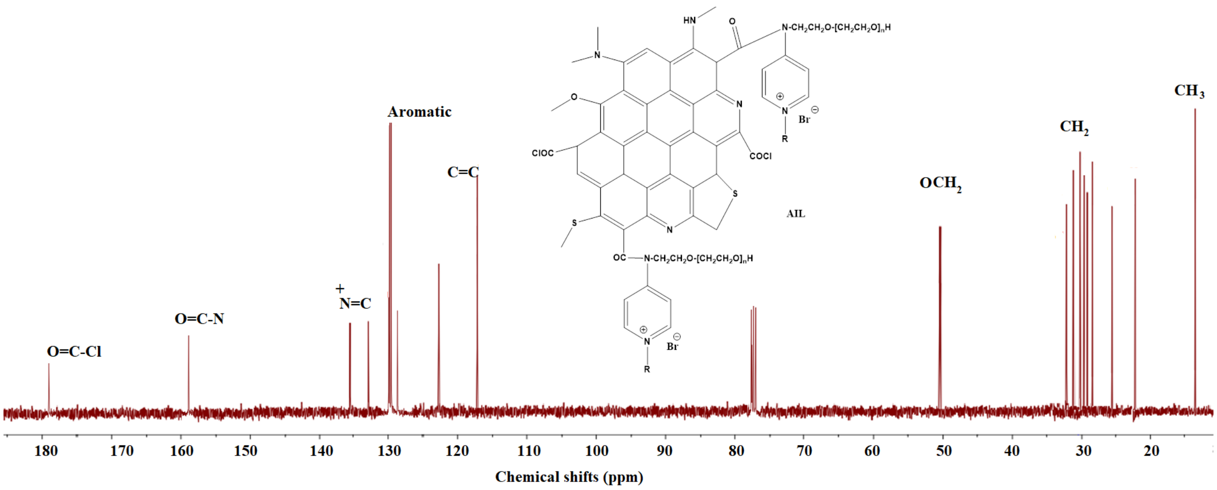

Fig. S3. ^{13}CNMR spectrum of AIL.
